# Supplementary material for: Efficacy and safety of veliparib combined with traditional chemotherapy for treating patients with lung cancer: a comprehensive review and meta-analysis
Source: PeerJ. 2023 Nov 10;11:e16402. doi: 10.7717/peerj.16402 (PMC10642362; doi:10.7717/peerj.16402)
Supplement: Supplemental Information 2 [file peerj-11-16402-s002.docx]

# Supplementary Materials

**Supplementary Table 1**. Search strategy

**1.Pubmed**

| Search number | Query | Results |
| --- | --- | --- |
| #1 | (((((((((((((((((((((((((((((((((((((((((((((((((((((((((((((((((((((((((((((((((((((((((((((Poly(ADP-ribose) Polymerase Inhibitors[MeSH Terms]) OR (olaparib [Supplementary Concept])) OR (niraparib [Supplementary Concept])) OR (mefuparib hydrochloride [Supplementary Concept])) OR (pamiparib [Supplementary Concept])) OR (stenoparib [Supplementary Concept])) OR (veliparib [Supplementary Concept])) OR (talazoparib [Supplementary Concept])) OR (rucaparib [Supplementary Concept])) OR (phenanthridone [Supplementary Concept])) OR (letermovir [Supplementary Concept])) OR (fluzoparib [Supplementary Concept])) OR (cofpropamine [Supplementary Concept])) OR (Poly(ADP-ribose) Polymerase Inhibitors[Title/Abstract])) OR (Inhibitors of Poly(ADP-ribose) Polymerase[Title/Abstract])) OR (PARP Inhibitor[Title/Abstract])) OR (Poly(ADP-Ribose) Polymerase Inhibitor[Title/Abstract])) OR (Poly(ADP-ribosylation) Inhibitor[Title/Abstract])) OR (NAD ADP ribosyltransferase inhibitor[Title/Abstract])) OR (PARS inhibitor[Title/Abstract])) OR (poly ADP ribose polymerase inhibitor[Title/Abstract])) OR (poly ADP ribose synthetase inhibitor[Title/Abstract])) OR (poly (ADP ribose) polymerase inhibitor[Title/Abstract])) OR (olaparib[Title/Abstract])) OR (AZD 2281[Title/Abstract])) OR (AZD2281[Title/Abstract])) OR (AZD-2281[Title/Abstract])) OR (AZD221[Title/Abstract])) OR (Lynparza[Title/Abstract])) OR (mk7339[Title/Abstract])) OR (mk 7339[Title/Abstract])) OR (ku 0059436[Title/Abstract])) OR (ku 59436[Title/Abstract])) OR (ku0059436[Title/Abstract])) OR (ku59436[Title/Abstract])) OR (niraparib[Title/Abstract])) OR (niraparib hydrochloride[Title/Abstract])) OR (Zejula[Title/Abstract])) OR (MK 4827[Title/Abstract])) OR (MK4827[Title/Abstract])) OR (MK-4827[Title/Abstract])) OR (niraparib tosilate[Title/Abstract])) OR (niraparib tosylate[Title/Abstract])) OR (zl 2306[Title/Abstract])) OR (zl2306[Title/Abstract])) OR (gsk 3985771[Title/Abstract])) OR (gsk3985771[Title/Abstract])) OR (jnj 64091742[Title/Abstract])) OR (jnj64091742[Title/Abstract])) OR (mefuparib[Title/Abstract])) OR (MPH1 cpd[Title/Abstract])) OR (pamiparib[Title/Abstract])) OR (BGB-290[Title/Abstract])) OR (bgb 290[Title/Abstract])) OR (bgb290[Title/Abstract])) OR (stenoparib[Title/Abstract])) OR (E7449[Title/Abstract])) OR (PARP inhibitor 2X-121[Title/Abstract])) OR (E-7449[Title/Abstract])) OR (veliparib[Title/Abstract])) OR (ABT 888[Title/Abstract])) OR (ABT888[Title/Abstract])) OR (ABT-888[Title/Abstract])) OR (talazoparib[Title/Abstract])) OR (Talzenna[Title/Abstract])) OR (BMN 673[Title/Abstract])) OR (BMN-673[Title/Abstract])) OR (BMN673[Title/Abstract])) OR (lt 006673[Title/Abstract])) OR (lt 673[Title/Abstract])) OR (lt006673[Title/Abstract])) OR (lt673[Title/Abstract])) OR (mdv 3800[Title/Abstract])) OR (mdv3800[Title/Abstract])) OR (rucaparib[Title/Abstract])) OR (PF-01367338[Title/Abstract])) OR (Rubraca[Title/Abstract])) OR (AG 014699[Title/Abstract])) OR (AG014699[Title/Abstract])) OR (AG-014699[Title/Abstract])) OR (co 338[Title/Abstract])) OR (co338[Title/Abstract])) OR (pf 01367338[Title/Abstract])) OR (pf 1367338[Title/Abstract])) OR (phenanthridone[Title/Abstract])) OR (letermovir[Title/Abstract])) OR (Prevymis[Title/Abstract])) OR (AIC246[Title/Abstract])) OR (fluzoparib[Title/Abstract])) OR (SHR3162[Title/Abstract])) OR (cofpropamine[Title/Abstract])) OR (senaparib[Title/Abstract])) OR (imp 4297[Title/Abstract])) OR (imp4297[Title/Abstract]) | 12834 |
| #2 | ((((((((((((((((((Lung Neoplasms[MeSH Terms]) OR (Small Cell Lung Carcinoma[MeSH Terms])) OR (Carcinoma, Non-Small-Cell Lung[MeSH Terms])) OR (Lung Neoplasms[Title/Abstract])) OR (Lung Neoplasm[Title/Abstract])) OR (Pulmonary Neoplasm[Title/Abstract])) OR (Lung Cancer[Title/Abstract])) OR (Pulmonary Cancer[Title/Abstract])) OR (Cancer of Lung[Title/Abstract])) OR (Small Cell Lung Carcinoma[Title/Abstract])) OR (Small Cell Cancer Of The Lung[Title/Abstract])) OR (Oat Cell Carcinoma of Lung[Title/Abstract])) OR (Oat Cell Lung Cancer[Title/Abstract])) OR (Small Cell Lung Cancer[Title/Abstract])) OR (Carcinoma, Non-Small-Cell Lung[Title/Abstract])) OR (Carcinoma, Non Small Cell Lung[Title/Abstract])) OR (Lung Carcinoma, Non-Small-Cell[Title/Abstract])) OR (Non Small Cell Lung Carcinoma[Title/Abstract])) OR (Nonsmall Cell Lung Cancer[Title/Abstract]) | 329940 |
| #3 | (((((((((((((((((((Lung Neoplasms[MeSH Terms]) OR (Small Cell Lung Carcinoma[MeSH Terms])) OR (Carcinoma, Non-Small-Cell Lung[MeSH Terms])) OR (Lung Neoplasms[Title/Abstract])) OR (Lung Neoplasm[Title/Abstract])) OR (Pulmonary Neoplasm[Title/Abstract])) OR (Lung Cancer[Title/Abstract])) OR (Pulmonary Cancer[Title/Abstract])) OR (Cancer of Lung[Title/Abstract])) OR (Small Cell Lung Carcinoma[Title/Abstract])) OR (Small Cell Cancer Of The Lung[Title/Abstract])) OR (Oat Cell Carcinoma of Lung[Title/Abstract])) OR (Oat Cell Lung Cancer[Title/Abstract])) OR (Small Cell Lung Cancer[Title/Abstract])) OR (Carcinoma, Non-Small-Cell Lung[Title/Abstract])) OR (Carcinoma, Non Small Cell Lung[Title/Abstract])) OR (Lung Carcinoma, Non-Small-Cell[Title/Abstract])) OR (Non Small Cell Lung Carcinoma[Title/Abstract])) OR (Nonsmall Cell Lung Cancer[Title/Abstract])) AND ((((((((((((((((((((((((((((((((((((((((((((((((((((((((((((((((((((((((((((((((((((((((((((((Poly(ADP-ribose) Polymerase Inhibitors[MeSH Terms]) OR (olaparib [Supplementary Concept])) OR (niraparib [Supplementary Concept])) OR (mefuparib hydrochloride [Supplementary Concept])) OR (pamiparib [Supplementary Concept])) OR (stenoparib [Supplementary Concept])) OR (veliparib [Supplementary Concept])) OR (talazoparib [Supplementary Concept])) OR (rucaparib [Supplementary Concept])) OR (phenanthridone [Supplementary Concept])) OR (letermovir [Supplementary Concept])) OR (fluzoparib [Supplementary Concept])) OR (cofpropamine [Supplementary Concept])) OR (Poly(ADP-ribose) Polymerase Inhibitors[Title/Abstract])) OR (Inhibitors of Poly(ADP-ribose) Polymerase[Title/Abstract])) OR (PARP Inhibitor[Title/Abstract])) OR (Poly(ADP-Ribose) Polymerase Inhibitor[Title/Abstract])) OR (Poly(ADP-ribosylation) Inhibitor[Title/Abstract])) OR (NAD ADP ribosyltransferase inhibitor[Title/Abstract])) OR (PARS inhibitor[Title/Abstract])) OR (poly ADP ribose polymerase inhibitor[Title/Abstract])) OR (poly ADP ribose synthetase inhibitor[Title/Abstract])) OR (poly (ADP ribose) polymerase inhibitor[Title/Abstract])) OR (olaparib[Title/Abstract])) OR (AZD 2281[Title/Abstract])) OR (AZD2281[Title/Abstract])) OR (AZD-2281[Title/Abstract])) OR (AZD221[Title/Abstract])) OR (Lynparza[Title/Abstract])) OR (mk7339[Title/Abstract])) OR (mk 7339[Title/Abstract])) OR (ku 0059436[Title/Abstract])) OR (ku 59436[Title/Abstract])) OR (ku0059436[Title/Abstract])) OR (ku59436[Title/Abstract])) OR (niraparib[Title/Abstract])) OR (niraparib hydrochloride[Title/Abstract])) OR (Zejula[Title/Abstract])) OR (MK 4827[Title/Abstract])) OR (MK4827[Title/Abstract])) OR (MK-4827[Title/Abstract])) OR (niraparib tosilate[Title/Abstract])) OR (niraparib tosylate[Title/Abstract])) OR (zl 2306[Title/Abstract])) OR (zl2306[Title/Abstract])) OR (gsk 3985771[Title/Abstract])) OR (gsk3985771[Title/Abstract])) OR (jnj 64091742[Title/Abstract])) OR (jnj64091742[Title/Abstract])) OR (mefuparib[Title/Abstract])) OR (MPH1 cpd[Title/Abstract])) OR (pamiparib[Title/Abstract])) OR (BGB-290[Title/Abstract])) OR (bgb 290[Title/Abstract])) OR (bgb290[Title/Abstract])) OR (stenoparib[Title/Abstract])) OR (E7449[Title/Abstract])) OR (PARP inhibitor 2X-121[Title/Abstract])) OR (E-7449[Title/Abstract])) OR (veliparib[Title/Abstract])) OR (ABT 888[Title/Abstract])) OR (ABT888[Title/Abstract])) OR (ABT-888[Title/Abstract])) OR (talazoparib[Title/Abstract])) OR (Talzenna[Title/Abstract])) OR (BMN 673[Title/Abstract])) OR (BMN-673[Title/Abstract])) OR (BMN673[Title/Abstract])) OR (lt 006673[Title/Abstract])) OR (lt 673[Title/Abstract])) OR (lt006673[Title/Abstract])) OR (lt673[Title/Abstract])) OR (mdv 3800[Title/Abstract])) OR (mdv3800[Title/Abstract])) OR (rucaparib[Title/Abstract])) OR (PF-01367338[Title/Abstract])) OR (Rubraca[Title/Abstract])) OR (AG 014699[Title/Abstract])) OR (AG014699[Title/Abstract])) OR (AG-014699[Title/Abstract])) OR (co 338[Title/Abstract])) OR (co338[Title/Abstract])) OR (pf 01367338[Title/Abstract])) OR (pf 1367338[Title/Abstract])) OR (phenanthridone[Title/Abstract])) OR (letermovir[Title/Abstract])) OR (Prevymis[Title/Abstract])) OR (AIC246[Title/Abstract])) OR (fluzoparib[Title/Abstract])) OR (SHR3162[Title/Abstract])) OR (cofpropamine[Title/Abstract])) OR (senaparib[Title/Abstract])) OR (imp 4297[Title/Abstract])) OR (imp4297[Title/Abstract])) | 533 |

**2.Cochrane**

| Search number | Query | Results |
| --- | --- | --- |
| #1 | MeSH descriptor: [Poly(ADP-ribose) Polymerase Inhibitors] explode all trees | 146 |
| #2 | (Poly(ADP-ribose) Polymerase Inhibitors):ti,ab,kw OR (olaparib):ti,ab,kw OR (niraparib):ti,ab,kw OR (mefuparib hydrochloride):ti,ab,kw OR (pamiparib):ti,ab,kw | 1214 |
| #3 | (stenoparib):ti,ab,kw OR (veliparib):ti,ab,kw OR (talazoparib):ti,ab,kw OR (rucaparib):ti,ab,kw OR (phenanthridone):ti,ab,kw | 521 |
| #4 | (fluzoparib):ti,ab,kw OR (letermovir):ti,ab,kw OR (cofpropamine):ti,ab,kw OR (Inhibitors of Poly(ADP-ribose) Polymerase):ti,ab,kw OR (PARP Inhibitor):ti,ab,kw | 845 |
| #5 | (Poly(ADP-Ribose) Polymerase Inhibitor):ti,ab,kw OR (Poly(ADP-ribosylation) Inhibitor):ti,ab,kw OR (NAD ADP ribosyltransferase inhibitor):ti,ab,kw OR (PARS inhibitor):ti,ab,kw OR (poly ADP ribose polymerase inhibitor):ti,ab,kw | 362 |
| #6 | (poly ADP ribose synthetase inhibitor):ti,ab,kw OR (poly (ADP ribose) polymerase inhibitor):ti,ab,kw OR (olaparib):ti,ab,kw OR (AZD 2281):ti,ab,kw OR (AZD2281):ti,ab,kw | 1075 |
| #7 | (AZD221):ti,ab,kw OR (Lynparza):ti,ab,kw OR (mk7339):ti,ab,kw OR (ku 0059436):ti,ab,kw OR (niraparib):ti,ab,kw | 333 |
| #8 | (niraparib hydrochloride):ti,ab,kw OR (Zejula):ti,ab,kw OR (MK4827):ti,ab,kw OR (niraparib tosilate):ti,ab,kw OR (niraparib tosylate):ti,ab,kw | 24 |
| #9 | (zl2306):ti,ab,kw OR (gsk3985771):ti,ab,kw OR (jnj64091742):ti,ab,kw OR (mefuparib):ti,ab,kw OR (MPH1 cpd):ti,ab,kw | 3 |
| #10 | (pamiparib):ti,ab,kw OR (bgb290):ti,ab,kw OR (stenoparib):ti,ab,kw OR (E7449):ti,ab,kw OR (PARP inhibitor 2X-121):ti,ab,kw | 7 |
| #11 | (veliparib):ti,ab,kw OR (ABT888):ti,ab,kw OR (talazoparib):ti,ab,kw OR (Talzenna):ti,ab,kw OR (BMN 673):ti,ab,kw | 358 |
| #12 | (lt673):ti,ab,kw OR (mdv3800):ti,ab,kw OR (rucaparib):ti,ab,kw OR (Rubraca):ti,ab,kw OR (PF-01367338):ti,ab,kw | 172 |
| #13 | (Rubraca):ti,ab,kw OR (AG014699):ti,ab,kw OR (co338):ti,ab,kw OR (pf 1367338):ti,ab,kw OR (phenanthridone):ti,ab,kw | 6 |
| #14 | (letermovir):ti,ab,kw OR (Prevymis):ti,ab,kw OR (AIC246):ti,ab,kw OR (fluzoparib):ti,ab,kw OR (SHR3162):ti,ab,kw | 81 |
| #15 | (cofpropamine):ti,ab,kw OR (senaparib):ti,ab,kw OR (imp4297):ti,ab,kw | 8 |
| #16 | #1 or #2 or #3 or #4 or #5 or #6 or #7 or #8 or #9 or #10 or #11 or #12 or #13 or #14 or #15 | 1780 |
| #17 | MeSH descriptor: [Lung Neoplasms] explode all trees | 10383 |
| #18 | MeSH descriptor: [Small Cell Lung Carcinoma] explode all trees | 536 |
| #19 | MeSH descriptor: [Carcinoma, Non-Small-Cell Lung] explode all trees | 5751 |
| #20 | (Lung Neoplasms):ti,ab,kw OR (Lung Neoplasm):ti,ab,kw OR (Pulmonary Neoplasm):ti,ab,kw OR (Lung Cancer):ti,ab,kw OR (Pulmonary Cancer):ti,ab,kw | 32544 |
| #21 | (Cancer of Lung):ti,ab,kw OR (Small Cell Lung Carcinoma):ti,ab,kw OR (Small Cell Cancer Of The Lung):ti,ab,kw OR (Oat Cell Carcinoma of Lung):ti,ab,kw OR (Oat Cell Lung Cancer):ti,ab,kw | 29547 |
| #22 | (Small Cell Lung Cancer):ti,ab,kw OR (Carcinoma, Non-Small-Cell Lung):ti,ab,kw OR (Carcinoma, Non Small Cell Lung):ti,ab,kw OR (Lung Carcinoma, Non-Small-Cell):ti,ab,kw OR (Non Small Cell Lung Carcinoma):ti,ab,kw | 18133 |
| #23 | (Nonsmall Cell Lung Cancer):ti,ab,kw | 10288 |
| #24 | #17 or #18 or #19 or #20 or #21 or #22 or #23 | 32958 |
| #25 | #16 and #24 | 168 |

**3.Embase**

| Search number | Query | Results |
| --- | --- | --- |
| #1 | poly AND 'adp ribose'/exp AND 'polymerase inhibitors' OR (poly:ti,ab,kw AND 'adp ribose':ti,ab,kw AND 'polymerase inhibitors':ti,ab,kw) OR 'mefuparib hydrochloride':ti,ab,kw OR ('inhibitors of poly':ti,ab,kw AND 'adp ribose':ti,ab,kw AND polymerase:ti,ab,kw) OR 'parp inhibitor':ti,ab,kw OR (poly:ti,ab,kw AND 'adp ribosylation':ti,ab,kw AND inhibitor:ti,ab,kw) OR 'nad adp ribosyltransferase inhibitor':ti,ab,kw OR 'pars inhibitor':ti,ab,kw OR 'poly adp ribose polymerase inhibitor':ti,ab,kw OR 'poly adp ribose synthetase inhibitor':ti,ab,kw OR (poly:ti,ab,kw AND 'adp ribose':ti,ab,kw AND 'polymerase inhibitor':ti,ab,kw) OR olaparib:ti,ab,kw OR 'azd 2281':ti,ab,kw OR azd2281:ti,ab,kw OR azd221:ti,ab,kw OR lynparza:ti,ab,kw OR mk7339:ti,ab,kw OR 'ku 0059436':ti,ab,kw OR niraparib:ti,ab,kw OR 'niraparib hydrochloride':ti,ab,kw OR zejula:ti,ab,kw OR mk4827:ti,ab,kw OR 'niraparib tosilate':ti,ab,kw OR 'niraparib tosylate':ti,ab,kw OR zl2306:ti,ab,kw OR gsk3985771:ti,ab,kw OR jnj64091742:ti,ab,kw OR mefuparib:ti,ab,kw OR 'mph1 cpd':ti,ab,kw OR pamiparib:ti,ab,kw OR bgb290:ti,ab,kw OR stenoparib:ti,ab,kw OR e7449:ti,ab,kw OR 'parp inhibitor 2x-121':ti,ab,kw OR veliparib:ti,ab,kw OR abt888:ti,ab,kw OR talazoparib:ti,ab,kw OR talzenna:ti,ab,kw OR 'bmn 673':ti,ab,kw OR lt673:ti,ab,kw OR mdv3800:ti,ab,kw OR rucaparib:ti,ab,kw OR 'pf 01367338':ti,ab,kw OR rubraca:ti,ab,kw OR ag014699:ti,ab,kw OR co338:ti,ab,kw OR 'pf 1367338':ti,ab,kw OR phenanthridone:ti,ab,kw OR letermovir:ti,ab,kw OR prevymis:ti,ab,kw OR aic246:ti,ab,kw OR fluzoparib:ti,ab,kw OR shr3162:ti,ab,kw OR cofpropamine:ti,ab,kw OR senaparib:ti,ab,kw OR imp4297:ti,ab,kw | 12423 |
| #2 | 'lung neoplasms'/exp OR 'small cell lung carcinoma'/exp OR 'carcinoma, non-small-cell lung'/exp OR 'lung neoplasms':ti,ab,kw OR 'lung neoplasm':ti,ab,kw OR 'pulmonary neoplasm':ti,ab,kw OR 'lung cancer':ti,ab,kw OR 'pulmonary cancer':ti,ab,kw OR 'cancer of lung':ti,ab,kw OR 'small cell lung carcinoma':ti,ab,kw OR 'small cell cancer of the lung':ti,ab,kw OR 'oat cell carcinoma of lung':ti,ab,kw OR 'oat cell lung cancer':ti,ab,kw OR 'small cell lung cancer':ti,ab,kw OR 'carcinoma, non-small-cell lung':ti,ab,kw OR 'carcinoma, non small cell lung':ti,ab,kw OR 'lung carcinoma, non-small-cell':ti,ab,kw OR 'non small cell lung carcinoma':ti,ab,kw OR 'nonsmall cell lung cancer':ti,ab,kw | 575010 |
| #3 | #1 AND #2 | 856 |

**4.Web of science**

| Search number | Query | Results |
| --- | --- | --- |
| #1 | Poly(ADP-ribose) Polymerase Inhibitors (Topic) or olaparib (Topic) or niraparib (Topic) or mefuparib hydrochloride (Topic) or pamiparib (Topic) or stenoparib (Topic) or veliparib (Topic) or talazoparib (Topic) or rucaparib (Topic) or phenanthridone (Topic) or letermovir (Topic) or fluzoparib (Topic) or cofpropamine (Topic) or Inhibitors of Poly(ADP-ribose) Polymerase (Topic) or PARP Inhibitor (Topic) or Poly(ADP-Ribose) Polymerase Inhibitor (Topic) or Poly(ADP-ribosylation) Inhibitor (Topic) or NAD ADP ribosyltransferase inhibitor (Topic) or PARS inhibitor (Topic) or poly ADP ribose polymerase inhibitor (Topic) or poly ADP ribose synthetase inhibitor (Topic) or poly (ADP ribose) polymerase inhibitor (Topic) or olaparib (Topic) or AZD 2281 (Topic) or AZD2281 (Topic) or AZD-2281 (Topic) or AZD221 (Topic) or Lynparza (Topic) or mk7339 (Topic) or mk 7339 (Topic) or ku 0059436 (Topic) or ku 59436 (Topic) or ku0059436 (Topic) or ku59436 (Topic) or niraparib (Topic) or niraparib hydrochloride (Topic) or Zejula (Topic) or MK 4827 (Topic) or MK4827 (Topic) or MK-4827 (Topic) or niraparib tosilate (Topic) or niraparib tosylate (Topic) or zl 2306 (Topic) or zl2306 (Topic) or gsk 3985771 (Topic) or gsk3985771 (Topic) or jnj 64091742 (Topic) or jnj64091742 (Topic) or mefuparib (Topic) or MPH1 cpd (Topic) or pamiparib (Topic) or BGB-290 (Topic) or bgb 290 (Topic) or bgb290 (Topic) or stenoparib (Topic) or E7449 (Topic) or PARP inhibitor 2X-121 (Topic) or E-7449 (Topic) or veliparib (Topic) or ABT 888 (Topic) or ABT888 (Topic) or ABT-888 (Topic) or talazoparib (Topic) or Talzenna (Topic) or BMN 673 (Topic) or BMN-673 (Topic) or BMN673 (Topic) or lt 006673 (Topic) or lt 673 (Topic) or lt006673 (Topic) or lt673 (Topic) or mdv 3800 (Topic) or mdv3800 (Topic) or rucaparib (Topic) or PF-01367338 (Topic) or Rubraca (Topic) or AG 014699 (Topic) or AG014699 (Topic) or AG-014699 (Topic) or co 338 (Topic) or co338 (Topic) or pf 01367338 (Topic) or pf 1367338 (Topic) or phenanthridone (Topic) or letermovir (Topic) or Prevymis (Topic) or AIC246 (Topic) or fluzoparib (Topic) or SHR3162 (Topic) or cofpropamine (Topic) or senaparib (Topic) or imp 4297 (Topic) or imp4297 (Topic) | 24732 |
| #2 | Lung Neoplasms (Topic) or Small Cell Lung Carcinoma (Topic) or Carcinoma, Non-Small-Cell Lung (Topic) or Lung Neoplasms (Topic) or Lung Neoplasm (Topic) or Pulmonary Neoplasm (Topic) or Lung Cancer (Topic) or Pulmonary Cancer (Topic) or Cancer of Lung (Topic) or Small Cell Lung Carcinoma (Topic) or Small Cell Cancer Of The Lung (Topic) or Oat Cell Carcinoma of Lung (Topic) or Oat Cell Lung Cancer (Topic) or Small Cell Lung Cancer (Topic) or Carcinoma, Non-Small-Cell Lung (Topic) or Carcinoma, Non Small Cell Lung (Topic) or Lung Carcinoma, Non-Small-Cell (Topic) or Non Small Cell Lung Carcinoma (Topic) or Nonsmall Cell Lung Cancer (Topic) | 439158 |
| #3 | #1 AND #2 | 1472 |

**
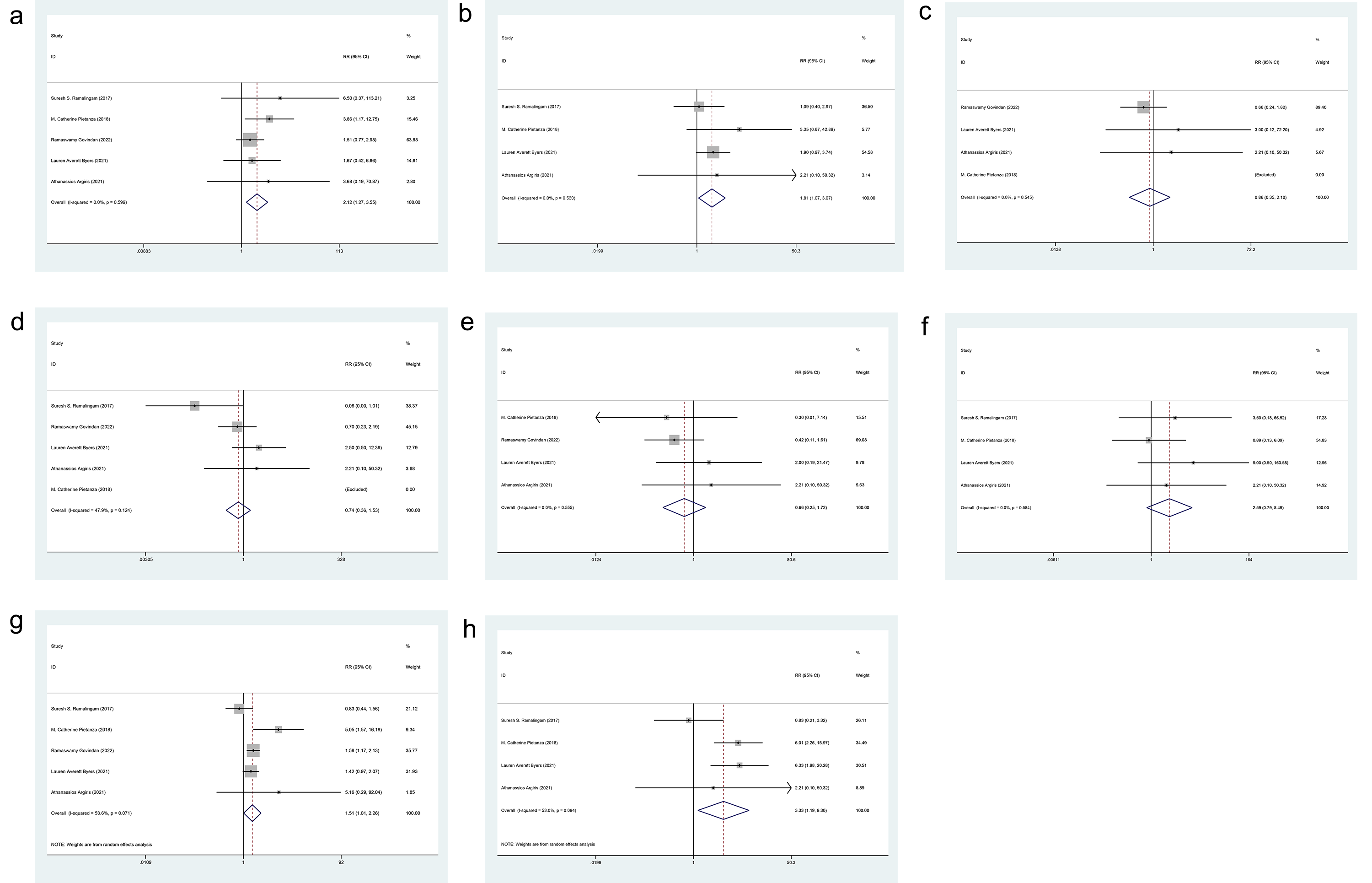
**

**Supplementary Figure1** Forest plot for safety of Veliparib combined with chemotherapy. a) Leukopenia; b) Anemia; c) Anorexia; d) Nausea; e) Vomiting; f) Fatigue; g) Neutropenia; h) Thrombocytopenia.
