# Supplementary material for: Efficacy and safety of veliparib combined with traditional chemotherapy for treating patients with lung cancer: a comprehensive review and meta-analysis
Source: PeerJ. 2023 Nov 10;11:e16402. doi: 10.7717/peerj.16402 (PMC10642362; doi:10.7717/peerj.16402)
Supplement: Supplemental Information 3 [file peerj-11-16402-s003.docx]

Systematic Review and/or Meta-Analysis Rationale

For systematic reviews / meta-analyses, authors need to provide the following information:

**1.Comment:** The rationale for conducting the systematic review / meta-analysis;

**Response:** Lung cancer accounts for 11.4% of all cancer cases and 18.0% of all cancer-related deaths throughout 2020, and overall treatment of advanced lung cancer is ineffective, with significant drug-related toxicity and a 5-year survival rate of only 10% - 20%.PARP inhibitors were initially developed with the aim of combining them with other therapies that damage the DNA of the cancer cell to weaken the cancer cell's ability to repair DNA damage to enhance the effectiveness of these therapies. However, in 2005, it was discovered that tumor cells carrying BRCA mutations were 1,000 times more sensitive to PARP inhibitors than tumor cells carrying the wild-type BRCA gene. And previous clinical trials have shown that the PARP inhibitor Veliparib in combination with simultaneous radiotherapy has achieved promising therapeutic results in NSCLC patients, however there may be toxicity associated with cytopenias. Therefore, this study evaluated the safety and efficacy of Veliparib in combination with chemotherapy and chemotherapy alone in the treatment of lung cancer patients by meta-analysis, aiming to provide evidence-based support for lung cancer treatment.

**2.Comment:** The contribution that it makes to knowledge in light of previously published related reports, including other meta-analyses and systematic reviews.

**Response:** This meta-analysis concluded by scientific statistical methods that Veliparib in combination with chemotherapy could improve pharmacogenomics-related PFS to some extent in lung cancer patients, but it did not improve OS and ORR, and there was a significant increase in adverse effects related to hematopoiesis. However, given the limited number of studies included in this review, further high-quality, multicenter randomized controlled trials are needed to confirm this conclusion.
